# Predicting salivary cortisol and sexual behavior stigma among MSM in the American Men’s Internet Survey 2019

**DOI:** 10.1038/s41598-023-44876-z

**Published:** 2023-10-23

**Authors:** Kate E. Dibble, Sarah M. Murray, Stefan D. Baral, Maria Zlotorzynska, John Mark Wiginton, Rob Stephenson, O. Winslow Edwards, Carrie Lyons, Jacob C. Rainey, Qian-Li Xue, Travis H. Sanchez

**Affiliations:** 1 Department of Epidemiology, Johns Hopkins Bloomberg School of Public Health, 615 N. Wolfe Street, Room E6133, Baltimore, MD 21205 USA; 2 Department of Mental Health, Johns Hopkins Bloomberg School of Public Health, Baltimore, MD 21205 USA; 3 Department of Epidemiology, Rollins School of Public Health, Emory University, Atlanta, GA 30322 USA; 4 Department of Health, Behavior and Society, Johns Hopkins Bloomberg School of Public Health, Baltimore, MD 21205 USA; 5 Department of Systems, Populations, and Leadership, School of Nursing, and The Center for Sexuality and Health Disparities, University of Michigan, Ann Arbor, MI 48109 USA; 6 Johns Hopkins School of Medicine, Baltimore, MD 21205 USA

**Keywords:** HIV infections, Epidemiology, Translational research, Risk factors

## Abstract

Physiological stress levels in response to sexual behavior stigma among men who have sex with men (MSM) in the United States (US) are understudied. The current study aims to explore the relationship between sexual behavior stigma and salivary cortisol both overall and stratified by race/ethnicity. If such an association exists, it may suggest that sexual behavior stigma can be physiologically measured or indicated by the presence of heightened salivary cortisol. A subsample of 667 MSM participants from the 2019 American Men’s Internet Survey (AMIS; *N* = 10,129) submitted morning (AM) and evening (PM) saliva cortisol samples using at-home mail-in collection kits. Average daily cortisol and daily cortisol change were calculated; simple linear regressions estimated associations between cortisol measures and sexual behavior stigma characterized in four different ways (ever and recent experience of individual stigma items; average ever and recent experience of three stigma scales: stigma from family and friends, anticipated healthcare stigma, general social stigma). Participants reported a mean age of 36.0 years (*SD* = 14.9), with most being non-Hispanic white (*n* = 480, 72.0%), Hispanic (*n* = 164, 12.3%), or Black/African American (*n* = 146, 10.9%), and identified as homosexual/gay (*n* = 562, 84.3%). Reporting ever experiencing healthcare providers gossiping was significantly associated with higher PM cortisol (*β* = 0.12, *p* = 0.001) and higher average daily cortisol (*β* = 0.11, *p* = 0.004), while reporting ever experiencing police refusing to protect was associated with higher AM cortisol (*β* = 0.08, *p* = 0.03) and higher average daily cortisol (*β* = 0.09, *p* = 0.02). Recent experiences of stigma were not significant predictors of any measure of cortisol. Measures of salivary cortisol may be used to characterize sexual behavior stigma among MSM populations, however more insight is needed to determine its exact relationship and strength.

## Introduction

Sexual behavior stigma is a social process that targets some members of society as less accepted than others based upon their sexual behaviors^1^. In the United States (US), one study has suggested that sexual behavior stigma such as verbal harassment, family gossip, and being afraid to be in public are prevalent at 56.7%, 50.0%, and 31.8%, respectively^2^. Men who have sex with men (MSM) experiencing such stigmas may exhaust coping resources, resulting in an increase of stress, possibily exacerbated by other intersectional factors such as racial/ethnic minority status^3–5^. Increased experiences of stigma lead to delayed medical care^6–8^, which furthers health deterioration and increased mortality^9–11^, categorizing this as a pertinent and ongoing public health issue.

Physiological acute and chronic stress have been documented as more prevalent among MSM compared to heterosexual men and have been associated with experienced sexual behavior stigma^12^. Past literature has highlighted the increasing prevalence of depression, suicidality, and substance use among US-based MSM populations in recent years influenced by the intersectional experiences of stigma. This can create a compounding effect on bodily responses such as stress, particularly among bisexual or older homosexual individuals, ethnic or racial minorities, and transgender men^13^. Yet, psychosocial support and mental health needs remain understudied^14,15^. Support services are underutilized within MSM populations due to physical and financial barriers^14,15^ and dissatisfaction with care often resulting from structural barriers (i.e., homophobia, heterosexism, etc.) due to sociocultural prejudicial norms and attitudes which are often compounded for racial/ethnic minorities^14,16^.

It is known that gay, bisexual, and other MSM in the US experience pervasive mental health disparities compared to heterosexual and cisgender peers^17^. These observed disparities have been explained through the minority stress framework, which posits that proximate (i.e., recent) and distal (i.e., ever) experiences of sexual behavior stigma, prejudice, and discrimination contribute to mental health symptomology^18^. The minority stress framework, therefore, suggests the existence of three experiential forms of minority stress among sexual minority populations: (1) external short- and long-term stressful events (such as stigma, sexual behavior disclosure, discrimination); (2) the expectation of such events; and (3) the internalization of negative societal attitudes^19,20^. MSM facing sexual behavior stigma remain disadvantaged, experiencing mental health issues such as depression, anxiety, and fatigue^21^ in addition to internalized homophobia and anti-gay and -LGB victimization that occur via daily stressors^22,23^.

One challenge in empirically establishing the role of stress is how to continue to accurately characterize this concept. Early methods of measuring stress were conducted subjectively via self-report, however recently, the validation of these measures has been at the forefront of many research agenda^24,25^. Past literature has suggested self-reported stress measures may not capture clear *objective* measures of stress, but rather *subjective*, remaining open to systematic error and gaps in validity^26^. Objectively, stress can be physiologically measured through plasma, hair, or saliva by capturing levels of cortisol, a hormone produced by the adrenal gland. Salivary cortisol samples and diurnal (highest cortisol upon waking) rhythmic analyses have been widely implemented in research and medical use alike to measure stress among MSM populations^23,27^. Literature has shown that social stressors (such as sexual behavior stigma and related discrimination, etc.) are linked to cortisol, depicting a positive correlation between these concepts^3,21,27–33^. Cortisol, therefore, has been utilized widely in recent years as a marker for future health and poor health outcomes such as the impact of stress, viral load, and CD4 counts relating to human immunodeficiency virus (HIV) infection among MSM^32,34–36^. Therefore, it is possible to infer that instead of utilizing *either* self-reported or physiological stress measures, it may be beneficial to use in tandem. It remains important, however, to explore this association with a temporal lens, as this topic has not been analyzed before. It also remains of interest to determine if there is a physiological link between the impact of acute or chronic experiences of sexual behavior stigma among the MSM in the US.

While links between biological measures of stress and self-reported poor HIV outcomes have been established^23^, avenues through which sexual behavior stigma may exacerbate physiological stress responses are not fully clear. There have been numerous studies providing insight into how measures of stress (via cortisol) predict self-reported health outcomes. Stress remains one of the leading causes of physical and mental health problems including cardiovascular disease^37^ and anxiety/depression^38^. Higher levels of cortisol due to prejudice, identity disclosure, and internalized stigma have been linked to multiple biological (such as physical health, immune response, CD4 count, viral load, and cardiovascular, metabolic, and hormonal issues)^23^ and psychological outcomes (such as anxiety, depression, suicidality, etc.)^12,28^. What research that does exist has found that sexual minority groups (including MSM) experience dysregulation in response to stress including perturbations of diurnal rhythms, due to ongoing daily minority stressors such as stigma^33,38^.

### Study objectives

The current descriptive study expands on what has been presented in past literature to examine the relationship between stress and sexual behavior stigma. Specifically, we sought to identify an exploratory relationship between stress (via salivary cortisol) and several types of sexual behavior stigma (stigma from family and friends, anticipated healthcare stigma, general social stigma) to understand its temporality (acute and chronic). We use the minority stress framework as a basis to assess the relationship between several types of sexual behavior stigma and salivary cortisol collected at-home by the participant as a proxy of stress among US MSM, which few studies have explored^12,22,23^. Various measures of cortisol (AM cortisol, PM cortisol, average daily cortisol, daily cortisol change) were obtained to ensure the validity of possible relationships between stress and sexual behavior stigma. We aimed to determine if there is an overall association between several types of sexual behavior stigma (anticipated, enacted, and perceived; ever and recent) and different salivary cortisol indicators. Secondarily, we also wanted to understand if there are differences in this association by race/ethnicity to further distinguish that sexual behavior stigma and stress (both acute and chronic) are an important intersectional experience among MSM^39,40^. We hypothesize that stress (via various measures of salivary cortisol) will be associated with sexual behavior stigma with more recent stigmatizing experiences, whereas racial/ethnic minority status will be indicative of higher stress.

## Method

### Recruitment and survey administration

The American Men’s Internet Survey (AMIS) aims to collect 10,000 complete surveys from eligible MSM annually. The primary goal of this repeated cross-sectional design is to monitor trends of human immunodeficiency virus (HIV) and sexually transmitted infection (STI) risk behavior, use of HIV and STI testing services, and access to other HIV and STI prevention and treatment services among US MSM. Data for the present analysis were taken from the 2019 cycle, which was implemented from September to December 2019. AMIS recruitment methods and procedural details have been published widely in past literature^41–44^. Eligibility criteria included being age 15 or older, reporting male sex at birth and male gender identity, residing in the US and providing a US ZIP code, reporting oral and/or anal sex with a male partner at least once in the past or identifying as gay or bisexual (sexual orientation was only considered for those aged 15–17). Eligible participants were asked to sign an electronic consent (e-consent) form or assent form for those aged 15–17 and completed an online questionnaire consisting of sociodemographic information, experiences of sexual behavior stigma, substance use, STI testing and diagnosis history, and the use of HIV preventive services. At survey conclusion, all participants were given the option of providing an email address for recontact as well as for the collection of biospecimen samples (e.g., salivary cortisol). All eligible and consented AMIS participants^45^ who provided an email address to be re-contacted for future studies were invited to participate in a substudy wherein they were asked to provide various biospecimens, including saliva, by mail. Those who consented and provided a valid mailing address were mailed a biospecimen self-collection kit, which included materials and instructions for two saliva samples, one to be taken within 30 min of waking (ante meridiem, AM) and one 30 min before going to sleep (post meridiem, PM). Participants mailed samples directly to Molecular Testing Laboratories in Vancouver, WA for processing using an enclosed prepaid mailer. Samples were then secondarily sent to Ayumetrix Laboratories in Lake Oswego, OR to conduct luminescence immunoassays to have them validated against the laboratory’s previously-validated immunoassay protocol. Samples were received between February and July 2020. Specimens were deidentified prior to mailing and were labelled with a unique participant identifier that could be linked back to the individual survey responses. No incentive was provided to the participants for completion of the survey, and participants who returned biospecimen samples for testing received a $50 gift card as compensation. The study was conducted in compliance with federal regulations governing the protection of human subjects and was reviewed and approved by Emory University’s Institutional Review Board (IRB#47676).

### Measures

#### Demographic information

Participants self-reported age, census region (Northeast, Midwest, South, West), race, ethnicity, education, annual income, sexual identity, and health insurance coverage at the time of survey. Due to invariability, data for demographic variables were dchotomized and included self-reported age (15–30, 30 or older), education (no college degree, college degree or higher), annual income based on the US Census Bureau^46^ as utilized in previous literature ($0–$39,999, $40,000 or more)^47^, and health insurance coverage (no coverage, coverage), as well as racial and ethnic identification (dummy variables for each of the following: Asian/Native Hawaiian/other Pacific Islander, non-Hispanic Black, Hispanic or Latino, non-Hispanic white [NHW], other/multiracial). These variables were condensed into one race/ethnicity variable for the purposes of analyses (NHW, Hispanic/Latino, non-Hispanic African American/Black, non-Hispanic other/multi-racial).

#### Sexual behavior stigma

Sexual behavior stigma was assessed using 13 items that asked participants if they had a specific stigma experience, each with three response options (no; yes, in the past 6 months; yes, but not in the past 6 months). Items for this scale were originally selected by applying a sociological framework to prior studies of HIV risks among MSM, identifying barriers in social capital and community services within numerous populations^48–50^. These 13 items as previously described^51,52^ make up three separate scales that assess different stigma domains: (1) stigma from family and friends (3 items [items 1–3]); (2) anticipated healthcare stigma (2 items [items 4–5]); and, (3) general social stigma (8 items; [items 6–13]) (see Table 1 for full item descriptions). For the current analysis, item responses were collapsed two ways: 1) no or yes to ever having the experience, and 2) no or yes to having the experience in the past 6 months. Internal consistency was adequate for all subscales (perceived stigma from family and friends: α = 0.70; anticipated healthcare stigma: α = 0.83; general social stigma: α = 0.70)^51^. Items for each scale were totaled for a score that could range from 0 to 3 for stigma from family and friends, 0–2 for anticipated healthcare stigma, and 0–8 for general social stigma. For analyses, we characterized these measures of sexual behavior stigma in four ways: (1) item-level stigma ever experienced (no, yes); (2) item-level recent stigma experienced (no, yes, within the past 6 months); (3) factor-level stigma ever experienced (totaled score on each factor); and (4) factor-level recent stigma experienced (totaled score on each sexual behavior stigma factor).

**Table 1:** Sexual behavior stigma scale items and responses by associated factors, American Mens’ Internet Survey, 2019.

Factors	Item	Item Description
Stigma from Family and Friends	1	Have you ever felt excluded from family activities because you have sex with men?
	2	Have you ever felt that family members have made discriminatory remarks or gossiped about you because you have sex with men?
	3	Have you ever felt rejected by your friends because you have sex with men?
Anticipated Healthcare Stigma	4	Have you ever felt afraid to go to healthcare services because you worry someone may learn you have sex with men?
	5	Have you ever avoided going to healthcare services because you worry someone may learn you have sex with men?
General Social Stigma	6	Have you ever felt that you were not treated well in a health center because someone knew that you have sex with men?
	7	Have you ever heard healthcare providers gossiping about you (talking about you) because you have sex with men?
	8	Have you ever felt that the police refused to protect you because you have sex with men?
	9	Have you ever felt scared to be in public places because you have sex with men?
	10	Have you ever been verbally harassed and felt it was because you have sex with men?
	11	Have you ever been blackmailed by someone because you have sex with men?
	12	Has someone ever physically hurt you (pushed, shoved, slapped, hit, kicked, choked, or otherwise physically hurt you)? [AND] Do you believe any of these experiences of physical violence was/were related to the fact that you have sex with men?
	13	Have you ever been forced to have sex when you did not want to (by forced, I mean physically forced, coerced to have sex, or penetrated with an object, when you did not want to)? [AND] Do you believe any of these experiences of sexual violence were related to the fact that you have sex with men?

Full reliability analyses and factor loadings are previously published^64^.

#### Remote salivary cortisol collection

Salivary cortisol collection has been utilized in previous literature to measure stress safely and effectively^30^. Several characterizations of salivary cortisol outcomes were included to better understand exploratory temporal associations with sexual behavior stigma, both overall and by race/ethnicity^12,53,54^. These characterizations included: (1) original AM cortisol; (2) original PM cortisol; (3) daily cortisol change $(\left| {PM cortisol - AM cortisol} \right|$); and (4) average daily cortisol ($\frac{{\left[ {AM cortisol + PM cortisol} \right]}}{2}$). For the purposes of the current study, original AM cortisol was measured to determine levels of stress hormone (cortisol) within 30 min upon waking, or the beginning of the daily diurnal cortisol cycle^55^. Original PM cortisol was measured to capture the end of diurnal cortisol cycle 30 min before going to sleep. Historically for salivary cortisol, average reference values for AM cortisol (cited between 7 am and 9 am) are 100–750 ng/dL and for PM cortisol (cited between 5 pm and 11 pm) are < 401 ng/dL^56^. Daily cortisol change $\left( {\left| {PM cortisol - AM cortisol} \right|} \right)$ measures the daily change in cortisol levels (in ng/dL) from AM to PM sample collections, also utilized in previous literature to determine the impact of daily stressors^33^, detect the effectiveness of stress reduction techniques^57^, and trends of diurnal rhythms longitudinally^58^. Lastly, average daily cortisol was calculated ($\frac{{\left[ {AM cortisol + PM cortisol} \right]}}{2}$) to conceptualize participants’ average stress due to daily stressors, which past research has utilized in measuring salivary cortisol among sexual minority populations^12,53^.

### Data analysis

The analytic sample was comprised of consented, eligible participants who reported oral and/or anal sex with a male partner in the past 12 months and who completed *both* morning (AM) and evening (PM) salivary cortisol samples who had not used corticosteriods. All analyses were conducted using a combination of SPSS for Windows version 27^59^ and Stata version 16^60^. Significance (*p*) values of less than 0.05 were considered significant across all analyses. All predictor and outcome variables were examined for outliers, normality, and missing data. Item-level missingness was imputed using Markov chain Monte Carlo (MCMC) multiple imputation using five iterations, utilizing all cortisol measures and stigma items as predictors. Sensitivity analyses were conducted to ensure no significant differences existed between associations from original and imputed data.

Descriptive analyses were conducted using chi-square tests for categorical variables and analyses of variance for continuous variables, both within the overall analytic sample and stratified by race/ethnicity. The current study utilized simple linear regressions with 95% confidence intervals (CIs) to determine significant associations between each cortisol outcome (AM cortisol, PM cortisol, daily cortisol change, average daily cortisol) and each sexual behavior stigma predictor (item [ever and recent]: 1–13; factors [ever and recent]: stigma from family and friends, anticipated healthcare stigma, general social stigma). If models were significant, race/ethnicity was analyzed as a possible stratification of these relationships. These models did not include covariates, as the current study was designed to be an exploratory analysis of the relationship (if any) between sexual behavior stigma items or factors and stress outcomes overall and by race/ethnic minority status.

### Ethics approval

The study was conducted in compliance with federal regulations governing the protection of human subjects and was reviewed and approved by Emory University’s Institutional Review Board (IRB#47676).

### Consent to participate

Informed consent was obtained from all participants included in the study.

## Results

### Participant characteristics

Participant characteristics for the total analytic sample are presented in Supplementary Table S1. The 2019 AMIS dataset included 10,129 MSM recruited online from across the US, 4275 (42.2%) provided email addresses for future contact and 1153 (26.9%) were screened for biospecimen sampling where 1068 (92.6%) consented. A total of 707 (61.3%) returned samples to the laboratory: 668 (94.4%) completed AM cortisol and 667 (94.3%) completed PM cortisol. The analytic sample included 667 AMIS participants (94.3%) who provided both AM and PM cortisol samples. On average, AM cortisol (*M* = 2.77, *SD* = 2.84) was significantly higher than PM cortisol (*M* = 1.64, *SD* = 2.92, *p* < 0.001). There were no statistical differences between the original and imputed dataset using the Pearson chi-square, likelihood ratio, or linear-by-linear association analyses.

### Sexual behavior stigma and cortisol

Ever experiencing some types of sexual behavior stigma was significantly associated with cortisol measures, shown in Table 2. Hypotheses were somewhat supported. Replying affirmatively to “Have you ever felt that the police refused to protect you because you have sex with men?” was associated with higher AM cortisol (mean difference = 0.088 units; 95% CI, 0.112–1.77; *p* = 0.026) compared to those who did not experience this stigma. Similarly, MSM who responded affirmatively to “Have you ever heard healthcare providers gossiping about you (talking about you) because you have sex with men?” had a PM cortisol score that was 0.128 units higher (95% CI 0.455–1.83, *p* = 0.001) that those who did not. Average cortisol was also found to be significantly predicted by replying affirmative to, “Have you ever heard healthcare providers gossip about you (talking about you) because you have sex with men?” (standardized *β* = 0.122, 95% CI 0.219–1.17, *p* = 0.004) and “Have you ever felt that the police refused to protect you because you have sex with men?” (Standardized *β* = 0.088, 95% CI 0.081–1.27, *p* = 0.026). No sexual behavior stigma items (ever or recent) predicted daily cortisol change. None of these relationships were significantly modified by any race/ethnicity category (data not shown).

**Table 2:** Cortisol differences among those who ever experienced sexual behavior stigma among 667 US men who have sex with men, American Mens’ Internet Survey, 2019.

Stigma	AM cortisol			PM cortisol			Average cortisol^a^			Daily cortisol change^b^		
	Standardized *β* (SE)	95% CI	*p*	Standardized *β* (SE)	95% CI	*p*	Standardized *β* (SE)	95% CI	*p*	Standardized *β* (SE)	95% CI	*p*
Excluded from family activities	− .055 (.236)	− .793 to .136	.165	.058 (.248)	− .126 to .847	.146	.005 (.171)	− .315 to .357	.902	− .021 (.251)	− .628 to .358	.590
Family made discriminatory remarks	.007 (.231)	− .414 to .494	.862	.013 (.246)	− .404 to .564	.746	.015 (.169)	− .270 to .392	.718	− .001 (.245)	− .487 to .477	.983
Rejected by friends	− .036 (.246)	− .705 to .259	.364	.022 (.255)	− .361 to .640	.584	− .008 (.176)	− .381 to .311	.843	− .022 (.259)	− .652 to .366	.581
Afraid to go to healthcare services	− .026 (.250)	− .661 to .321	.497	− .046 (.257)	− .806 to .202	.240	− .052 (.178)	− .589 to .109	.177	− .037 (.263)	− .764 to .268	.345
Avoided going to healthcare services	− .011 (.277)	− .619 to .468	.785	− .008 (.284)	− .615 to .498	.837	− .014 (.197)	− .456 to .317	.724	.002 (.291)	− .555 to .587	.956
Not treated well in healthcare center	.047 (.511)	− .397–1.60	.236	.021 (.526)	− .758 to 1.30	.601	.048 (.365)	− .272 to 1.16	.224	− .017 (.538)	− 1.28 to .828	.671
Healthcare providers gossiping about you	.028 (.341)	− .425 to .915	.472	**.128 (.351)**	**.455 ** to **1.83**	**.001**	**.112 (.244)**	**.219** to **1.17**	**.004**	.021 (.361)	− .515 to .902	.592
Police refused to protect you	**.088 (.423)**	**.112** to **1.77**	**.026**	.037 (.441)	− .458 to 1.27	.355	**.088 (.305)**	**.081** to **1.27**	**.026**	.053 (.449)	− .280 to 1.48	.181
Scared to be in public places	.006 (.224)	− .402 to .476	.868	.059 (.230)	− .107 to .795	.135	.044 (.159)	− .131 to .493	.255	.036 (.236)	− .247 to .680	.359
Verbally harassed	.024 (.222)	− .301 to .572	.543	.025 (.228)	− .302 to .593	.524	.034 (.158)	− .172 to .447	.384	.017 (.234)	− .361 to .559	.672
Blackmailed	− .041 (.327)	− .983 to .301	.297	− .015 (.338)	− .796 to .532	.697	− .039 (.233)	− .690 to .227	.322	− .059 (.344)	− 1.19 to .155	.131
Ever physically hurt you because MSM	− .004 (.230)	− .472 to .430	.928	− .029 (.237)	− .641 to .288	.456	− .022 (.164)	− .415 to .229	.571	.013 (.242)	− .395 to .557	.737
Forced to have sex because MSM	.005 (.253)	− .464 to .529	.898	.043 (.266)	− .231 to .815	.273	.034 (.183)	− .198 to .521	.379	.041 (.268)	− .243 to .808	.292

*MSM* men who have sex with men, *β* coefficient from linear regression for difference in cortisol for those who ever experienced stigma compared to those who never did; calculated automatically via Stata, version 16. *SE* standard error.

^a^Cortisol average = AM cortisol + PM cortisol/2.

^b^Daily cortisol change =|PM cortisol – AM cortisol|.

“Ever” indicates ever experiencing the sexual behavior stigma item at some point in one’s life.

Referent group was those who never experienced stigma.

Bold font indicates statistical significance (*p* < .05).

Full item descriptions can be found in Table 1.

The second portion of our hypothesis regarding experiencing recent stigma was not supported. None of the sexual behavior stigma items regarding recent stigma experiences (within the past six months) significantly predicted any cortisol measure. These findings are depicted in Supplementary Table S2.

Neither ever nor recent (within the past six months) experience(s) regarding the three sexual behavior stigma factors (stigma from family and friends, anticipated healthcare stigma, general social stigma) significantly predicted any cortisol measure (data not shown).

### Stratification by race/ethnicity

Most participants were NHW (72.0%), Hispanic (12.3%), or non-Hispanic Black/African American (7.5%). More details are depicted in Supplementary Table S1. Overall, there was no significant variation in stigma item endorsement or cortisol outcomes by race/ethnicity. The frequency of item-level sexual behavior stigma experience (ever, recent) both overall and by race/ethnicity are depicted in Table 3. There existed few differences among participants responding affirmatively to, “Have you ever felt excluded from family activities because you have sex with men?” (*p* = 0.03), “Have you ever been verbally harassed and felt it was because you have sex with men?” (*p* = 0.001), and “Has someone ever physically hurt you (pushed, shoved, slapped, hit, kicked, choked, or otherwise physically hurt you)?” *and* “Do you believe any of these experiences of physical violence was/were related to the fact that you have sex with men? (*p* = 0.007), where it was more common among NHW MSM than minority racial/ethnic groups. It is important to note however, that the proportion of racial/ethnic minority MSM in the analytic sample is limited. Among recent experiences of sexual behavior stigma, answering affirmatively to, “Have you felt excluded from family activities because you have sex with men?” occurred more often among MSM identifying as “other” racial/ethnic groups (*p* = 0.001). The average sexual behavior stigma factor score (ever, recent) and salivary cortisol outcome overall and by race/ethnicity are shown in Table 4. There were no significant differences in average of sexual behavior stigma factors (ever, recent) except for among stigma from family (recent), where average scores were significantly higher among MSM identifying with “other” racial/ethnic minority groups. There were no significant differences among any cortisol outcome and race/ethnicity.

**Table 3:** Experience of sexual behavior stigma by item (ever, recent), overall and by race/ethnicity.

Characteristics	Race/ethnicity					
	Total (N = 667) *n* (%)	NHW (N = 480) *n* (%)	Hispanic/latino (N = 82) *n* (%)	AA/NHB (N = 50) *n* (%)	Other/multi-racial^a^ (N = 54) *n* (%)	*p* value
Item-level sexual behavior stigma experiences (ever)						
Excluded from family activities	227 (36.0)	177 (38.7)	18 (23.1)	12 (26.7)	20 (40.0)	**0.03**
Family made discriminatory remarks	309 (50.6)	227 (51.4)	36 (48.6)	21 (44.7)	25 (52.1)	0.82
Rejected by friends	195 (30.4)	149 (32.2)	15 (18.3)	13 (27.1)	18 (36.0)	0.07
Afraid to go to healthcare services	178 (26.8)	134 (28.0)	17 (20.7)	12 (24.0)	15 (28.3)	0.53
Avoided going to healthcare services	133 (20.0)	94 (19.7)	14 (17.1)	11 (22.0)	14 (25.9)	0.62
Not treated well in healthcare center	33 (5.1)	27 (5.8)	1 (1.3)	3 (6.0)	2 (3.8)	0.36
Healthcare providers gossiping about you	79 (12.2)	63 (13.5)	6 (7.3)	5 (10.0)	5 (9.4)	0.44
Police refused to protect you	49 (7.7)	36 (7.8)	6 (7.3)	5 (10.6)	2 (4.1)	0.68
Scared to be in public places	289 (43.9)	213 (44.7)	36 (44.4)	14 (29.2)	25 (48.1)	0.19
Verbally harassed	324 (49.2)	256 (53.7)	32 (39.5)	13 (26.5)	23 (45.1)	**0.001**
Blackmailed	87 (13.1)	60 (12.6)	8 (9.8)	8 (16.0)	11 (20.8)	0.25
Ever physically hurt you because MSM	241 (36.3)	192 (40.3)	23 (28.0)	13 (26.0)	12 (22.6)	**0.007**
Forced to have sex because MSM	154 (25.0)	115 (24.5)	24 (29.3)	9 (18.0)	15 (28.8)	0.76
Item-level sexual behavior stigma experiences (recent)						
Excluded from family activities	96 (15.2)	70 (15.3)	5 (6.4)	5 (10.0)	16 (32.0)	**0.001**
Family made discriminatory remarks	136 (22.3)	97 (21.9)	18 (24.3)	5 (10.6)	16 (33.3)	0.06
Rejected by friends	48 (7.5)	34 (7.3)	5 (6.3)	3 (6.3)	6 (11.1)	0.62
Afraid to go to healthcare services	59 (8.9)	42 (8.8)	7 (8.5)	6 (12.0)	4 (7.5)	0.86
Avoided going to healthcare services	37 (5.6)	25 (5.2)	4 (4.9)	3 (6.0)	5 (9.3)	0.66
Not treated well in healthcare center	10 (1.5)	8 (1.7)	0 (0.0)	1 (2.0)	1 (1.9)	0.69
Healthcare providers gossiping about you	18 (2.8)	12 (2.6)	3 (3.9)	2 (4.1)	1 (1.9)	0.82
Police refused to protect you	4 (0.6)	3 (0.7)	0 (0.0)	1 (2.1)	0 (0.0)	0.47
Scared to be in public places	138 (20.9)	97 (20.3)	18 (22.2)	8 (16.7)	15 (28.8)	0.44
Verbally harassed	78 (11.8)	61 (12.8)	10 (12.3)	1 (2.0)	6 (11.8)	0.17
Blackmailed	10 (1.5)	7 (1.5)	0 (0.0)	2 (4.0)	1 (1.9)	0.33
Ever physically hurt you because MSM	22 (3.3)	16 (3.4)	3 (3.7)	0 (0.0)	3 (5.7)	0.44
Forced to have sex because MSM	22 (3.4)	15 (3.2)	4 (4.9)	1 (2.0)	2 (3.7)	0.81

*AA* African American, *MSM*  men who have sex with men, *NHB* non-hispanic black, *NHW* non-hispanic white.

^a^Includes Asian/Native Hawaiian or Other Pacific Islander, unknown, and multi-racial groups.

“Ever” indicates ever experiencing the sexual behavior stigma factor at some point in one’s life.

“Recent” indicates recently (within the past 6 months) experiencing the sexual behavior stigma item or factor.

Bold font indicates significant group differences by race/ethnicity via chi-square test of independence (*p* < .05).

Full item descriptions can be found in Table 1.

**Table 4:** Sexual behavior stigma by factor (ever, recent) and physiological cortisol averages, overall and by race/ethnicity.

Characteristics	Race/ethnicity					
	Total (N = 667) M (*SD*)	NHW (N = 480) M (*SD*)	Hispanic/latino (N = 82) M (*SD*)	AA/NHB (N = 50) M (*SD*)	Other/multi-racial^a^ (N = 54) M (*SD*)	*p* value
Sexual behavior stigma factors						
Stigma from family—ever	1.16 (1.14)	1.21 (1.17)	0.90 (0.93)	1.00 (1.08)	1.26 (1.17)	0.11
Stigma from family—recent	0.44 (0.80)	0.44 (0.81)	0.36 (0.61)	0.28 (0.62)	0.80 (0.94)	**0.01**
Anticipated healthcare stigma—ever	0.46 (0.78)	0.47 (0.77)	0.37 (0.74)	0.46 (0.81)	0.52 (0.84)	0.68
Anticipated healthcare stigma—recent	0.14 (0.47)	0.14 (0.46)	0.13 (0.46)	0.18 (0.52)	0.15 (0.53)	0.95
General social stigma—ever	1.93 (1.72)	1.96 (1.72)	1.67 (1.65)	1.42 (1.61)	1.78 (1.79)	0.15
General social stigma—recent	0.46 (0.84)	0.46 (0.83)	0.47 (0.89)	0.26 (0.65)	0.51 (0.77)	0.45
Cortisol						
AM cortisol	2.77 (2.83)	2.86 (2.66)	2.17 (1.97)	3.14 (5.23)	2.49 (2.12)	0.14
PM cortisol	1.64 (2.91)	1.61 (3.07)	1.62 (2.61)	1.56 (1.88)	2.01 (2.74)	0.81
Cortisol flatness^b^	2.97 (2.99)	3.05 (2.84)	2.64 (2.42)	2.85 (5.17)	2.99 (2.39)	0.71
Average cortisol^c^	2.20 (2.02)	2.23 (2.07)	1.89 (1.46)	2.35 (2.70)	2.23 (1.54)	0.51

*AA* African American, *NHB* non-hispanic black, *NHW* non-hispanic white.

^a^Includes Asian/Native Hawaiian or Other Pacific Islander, unknown, and multi-racial groups.

^b^Cortisol flatness =|PM cortisol – AM cortisol|.

^c^Cortisol average = AM cortisol + PM cortisol/2.

“Ever” indicates ever experiencing the sexual behavior stigma factor at some point in one’s life.

“Recent” indicates recently (within the past 6 months) experiencing the sexual behavior-based stigma item or factor.

Bold font indicates significant group mean differences by race/ethnicity via one-way analysis of variance (*p* < .05).

## Discussion

The purpose of this descriptive, feasibility study was to begin to characterize the relationship between cortisol measured stress and sexual behavior stigma (ever and recently experienced)^2^. Our exploratory hypothesis that stress across various measures of cortisol would be associated with all measures of sexual behavior stigma, with more recent stigmatizing experiences more strongly associated with higher stress was somewhat indicated. Specifically, some sexual behavior stigma items indicating ever experiencing stigma predicted various measures of cortisol. In our sample, cortisol levels from AM samples appeared to be significantly higher compared to PM collection, similar to previous literature which has established that cortisol levels are most often highest in the morning upon waking^55,61^. There remained consistent relationships between stress levels and ever experiencing various instances of sexual behavior stigma, which may reinforce the use of salivary cortisol for measuring stress among populations of US-based MSM.

Cortisol was positively associated with reporting certain experiences of sexual behavior stigma, including feeling that the police refused to provide protection because of one’s sexual behavior with other men (AM cortisol, average cortisol) and having ever heard healthcare providers gossiping about oneself because of one’s engagement in sexual behavior with other men (PM cortisol, average cortisol). These two experiences have in common their occurrence during interactions with institutions, specifically the healthcare system and law enforcement. These relationships were not significantly modified by race/ethnicity; although we did not see differences here, it does not mean that structural racism or homonegativity are not present^39,62,63^. The actual events that led participants to seek police assistance and/or protection may also be contributing to the stress experienced. Problematically, there remains lack of training for medical professionals concerning LGBTQ + health^2^. Stigma within healthcare settings has also been shown to be prevalent among MSM in the US^64^ and may also increase stress through possible barriers in obtaining appropriate healthcare suggesting nonadherence and mistrust^65^.

It is possible that these instances of stigmatizing behavior from police and medical professionals are traumatizing, elevating stress, as previously suggested^20^, or better predict impacts of structural stigma more broadly. On the other hand, it remains plausible that this effect is chronic and therefore decreases the ability for stress changes to be determined. For instance, experiencing high levels of chronic stress over time may dehabituate smaller stressors, indicative of the nonsignificance within a measure of cortisol via salivary collection^12^. Thus, those with more recent sexual behavior stigma experiences have an attenuated cortisol response because they may be living in a continued stress response and therefore appear similar to those without sexual behavior stigma experiences. Among those with older sexual behavior stigma experiences, it remains possible that they no longer have an attenuated cortisol response due to resiliency^66^, but they remain primed to have larger stress response increases due to other stressors compared to those who have never experienced sexual behavior stigma.

Previous research has long pushed for legal and policy reform to improve protective laws for subgroups of MSM such as those outlined in the current study^67^. While there exists structural barriers to healthcare access and the perception of safety among some MSM, such as legislative protections, it is evident that these barriers should be addressed, as sexual behavior stigma and structural barriers at both individual- and community-levels are associated with individual-level determinants of HIV and sexually transmitted infection risk among MSM^2,68^. Thus, the structural and social barriers that persist among MSM may exacerbate stress responses leading to stress habituation^12^. Previous research has suggested political and judiciary rectification to enhance inclusivity and safeguards against sexual identity discrimination^69^, therefore suggesting that stress among this population would decrease as well. Others have suggested that increasing awareness through education and training among police and healthcare professionals, generally, have been beneficial but require additional social activism to further assist these groups^69^, working toward the same goal. Sexual behavior stigma and its relationship with acute and chronic stress, however, remains complex, requiring intervention on multiple levels of legislative and sociocultural environments to begin to address these concerns.

Report of recent experiences (within the past six months) of each act or form of stigma were not associated with any measure of stress. A possible explanation for this may be resilience. For example, Austin and colleagues^29^ found among young adult sexual minorities in the US, there were no differences in diurnal salivary cortisol levels across sexual identities possibly due to developing coping strategies that protect them against the deleterious effects of stigma^20,29^. Thus, all associations between sexual behavior stigma items and measures of stress within the current study may have nonsignificant findings because participants were used to experiencing stress and sexual behavior stigma, and therefore, their stress reactivity are skewed, especially in relation to healthcare and law enforcement. It may be possible that in fact, their diurnal cortisol rhythms are skewed, leading to the association between ever experiencing sexual behavior stigma and any cortisol outcome^70^. It appears as though older sexual behavior stigma experiences fit this model even more so, but differences were found within the current study that suggest otherwise. This potential explanation may also apply to the finding outlined above, that average cortisol was significantly predicted by both ever feeling that the police refused to provide protection because of engaging in sexual behavior with other men and ever hearing healthcare professionals gossip because of engaging in sexual behavior with other men, but not other items related to sexual behavior stigma. As a result, future studies should continue to examine incidences of sexual behavior stigma and measures of stress, both acute and chronic, to further determine the pattern and relationship between them. Simiarly, the current study only examined one source of stress (sexual behavior stigma), however, there exist numerous others that may be imperative to assess relative to the cortisol-sexual behavior stigma relationship to better discern possible confounding factors.

### Limitations

The results of the current study should be interpreted with consideration of its limitations. As participants were recruited via convenience sampling methods, findings are not generalizable to all US-based MSM or to all MSM with online access^71^. The sample that did not provide cortisol data was similar in race/ethnicity, age, education, US region, income, and sexual identity, introducing the potential for selection bias. The sample was largely homogeneous in terms of race/ethnicity and sexual identity and was highly educated, further limiting generalizability. Despite our intent to stratify by race/ethnicity, subgroup sizes were limited therefore affecting generalizability and the power to determine statistically significant associations. We also utilized simple predictors in a dichotomous nature (i.e., ever experiencing stigma in the past six months, etc.) to determine if there was a relationship between cortisol and experiencing sexual behavior stigma. It is possible that analyzing sexual behavior stigma’s relationship with cortisol this way is too simple and may have potentially omitted cortisol’s tendency to change over time. Future analyses including these variables should include cortisol’s ability to ebb and flow over time. The study is cross-sectional and does not allow for causal inferences to be made about the relationships between sexual behavior stigma and stress. The use of salivary cortisol may not be able to account for the relationship between stress, instances of sexual behavior stigma, and potential chronic stress, which is exceedingly difficult to define, since chronic stress can also appear as different diurnal patterns^29,58^. The current study also did not control for daily or current stressors (or the lack thereof) in close proximity to cortisol collection. Even though similar experiences of stigma did not predict all measures of cortisol (e.g., AM, PM, average, etc.), we know these levels vary throughout the day. It is possible that inaccuracies in cortisol collection occurred or that some measures of cortisol should be used in place of others. As reported previously^72^, cortisol samples taken over multiple days at the same times of day would be more beneficial in predicting patterns. However, the main purpose of this AMIS cycle was not to collect cortisol, as it was exploratory in nature for future study implementation, and collecting cortisol samples among this hard-to-reach population is important to report and should be expanded upon in future research. Sexual behavior stigma items assessed experiences within the past six months and ever, which can introduce temporal recall bias. Procedurally, issues relating to cortisol collection, preservation, and analysis that affect accuracy remain possible. For instance, it is possible that participants did not collect salivary cortisol samples within 30 min of waking and 30 min before sleep. It also remains possible that contamination occurred during transport or medication contamination such as the use of acidic substances and blood in the mouth can alter cortisol levels. Lastly, laboratory cortisol levels and how others compare to other external benchmarks exists and varies^73^.

Also, it could be possible that participants experienced sexual behavior stigma after completion of the online survey measure but before cortisol collection and that distress symptoms changed between the time of survey assessment and biospecimen self-collection. Therefore, it may be possible that we did not capture recent enough or nuanced experiences of certain types of sexual behavior stigma or stress to show necessary sensitivity in predicting the outcomes. As an exploratory study, these analyses did not account for potential confounders or post-hoc subgroup analyses, which may have influenced the relationships examined and interpreted. There may exist limitations in how sexual behavior stigma was measured, as stigma from family and friends was comprised of three items, and anticipated healthcare stigma of only two, limiting the scope of their applications.

Therefore, future research should expand on the exploratory results provided in the current study by focusing on recency, magnitude, and type of sexual behavior stigma experienced, as well as capturing measures of perceived stress to determine a true baseline to analyze physiological cortisol. Additionally, collecting more than two samples per day across multiple days, using more concrete increments at sample collection may absolve variability. Utilizing these measures in a larger, more diverse sample would be more beneficial in determining patterns across racial/ethnic groups, as suggested in the current study.

## Conclusion

This research contributes to the growing literature on linking experiences of sexual behavior stigma with biological stress processes among MSM. Significant findings, though few, underscore the consistent nature of sexual behavior stigma, evident in the physiological manifestations of its impact and in the fact that the specific experiences may be linked to manifestations multifactorial processes. There is a particular susceptibility to the effects of sexual behavior stigma during times of heightened vulnerability to harm, when seeking help and care from institutions established for such purposes—and when one is perhaps most likely to assume their sexuality is inconsequential to their receipt of help—is thwarted due to anti-MSM prejudice. Additional research is needed to delineate further how these particular sexual behavior stigmas affect both stress processes and more distal health outcomes among MSM. Intervention development focused on accessible, affirmative medical, and mental healthcare services to address stress and stress hormone reactivity for MSM warrant particular attention.

### Supplementary Information

Supplementary Table S1.

Supplementary Table S2.

## Data Availability

The dataset(s) generated and/or analyzed during the current study are available at the Principal Investigator (PI)’s discretion upon reasonable request.

## Code availability

Syntax is available upon reasonable request from the corresponding author.
