# Supplementary material for: Predicting salivary cortisol and sexual behavior stigma among MSM in the American Men’s Internet Survey 2019
Source: Sci Rep. 2023 Oct 23;13:18082. doi: 10.1038/s41598-023-44876-z (PMC10593931; doi:10.1038/s41598-023-44876-z)
Supplement: Supplementary file 1 — Supplementary Table S1. [file 41598_2023_44876_MOESM1_ESM.docx]

| **Supplementary Table S1.** Participant characteristics, overall and stratified by race/ethnicity, 667 US men who have sex with men, American Men’s Internet Survey, 2019 | | | | | |
| --- | --- | --- | --- | --- | --- |
| **Characteristics** | **TOTAL** | **RACE/ETHNICITY** | | | |
|  | **No. (%)**  **(N=667)** | **NHW**  **(N=480)** | **Hispanic/Latino**  **(N=82)** | **AA/NHB**  **(N=50)** | **Other/Multi-Racial***  **(N=54)** |
| **Race/ethnicity** | |  |  |  |  |
| Asian/Native Hawaiian/OPI* | 29 (4.3) | - | - | - | - |
| NHB | 50 (7.5) | - | - | - | - |
| Hispanic/Latino | 82 (12.3) | - | - | - | - |
| NHW | 480 (72.0) | - | - | - | - |
| Other/Multi-racial | 25 (3.7) | - | - | - | - |
| Missing | 1 (0.1) | - | - | - | - |
| **Sexual Identity** | |  |  |  |  |
| Heterosexual or straight | 3 (0.4) | 2 (0.4) | 0 (0.0) | 1 (2.0) | 0 (0.0) |
| Homosexual or gay | 562 (84.3) | 410 (85.4) | 67 (81.7) | 38 (76.0) | 46 (85.2) |
| Bisexual | 86 (12.9) | 59 (12.3) | 11 (13.4) | 9 (18.0) | 7 (13.0) |
| Another sexual identity | 12 (1.8) | 6 (1.3) | 4 (4.9) | 1 (2.0) | 1 (1.9) |
| Missing | 4 (0.5) | 3 (0.6) | 0 (0.0) | 1 (2.0) | 0 (0.0) |
| **Education** | |  |  |  |  |
| No college degree | 274 (41.1) | 184 (38.3) | 41 (50.0) | 29 (58.0) | 20 (37.0) |
| College degree or higher | 393 (58.9) | 296 (61.7) | 41 (50.0) | 21 (42.0) | 34 (63.0) |
| Missing | 0 (0.0) | 0 (0.0) | 0 (0.0) | 0 (0.0) | 0 (0.0) |
| **Age** | |  |  |  |  |
| Younger than 30 years | 307 (46.0) | 191 (39.8) | 62 (75.6) | 25 (50.0) | 29 (53.7) |
| 30 years or older | 360 (54.0) | 289 (60.2) | 20 (24.4) | 25 (50.0) | 25 (46.3) |
| Missing | 0 (0.0) | 0 (0.0) | 0 (0.0) | 0 (0.0) | 0 (0.0) |
| **Annual income** | |  |  |  |  |
| $0 - $39,999 | 206 (30.9) | 138 (28.7) | 32 (39.0) | 23 (46.0) | 13 (24.1) |
| $40,000 or more | 422 (63.3) | 321 (66.9) | 42 (51.2) | 21 (42.0) | 37 (68.5) |
| Missing | 39 (5.8) | 21 (4.4) | 8 (9.8) | 6 (12.0) | 4 (7.4) |
| **Census region** | |  |  |  |  |
| Northeast | 123 (18.4) | 92 (19.2) | 11 (13.4) | 5 (10.0) | 15 (27.8) |
| Midwest | 133 (19.9) | 110 (22.9) | 10 (12.2) | 8 (16.0) | 5 (9.3) |
| South | 264 (39.6) | 178 (37.1) | 34 (41.5) | 35 (70.0) | 16 (29.6) |
| West | 147 (22.0) | 100 (20.8) | 27 (32.9) | 2 (4.0) | 18 (33.3) |
| Missing | 0 (0.0) | 0 (0.0) | 0 (0.0) | 0 (0.0) | 0 (0.0) |
| **HIV status** | |  |  |  |  |
| Positive | 52 (7.8) | 37 (7.7) | 4 (4.9) | 10 (20.0) | 1 (1.9) |
| Negative | 615 (92.2) | 443 (92.3) | 78 (95.1) | 40 (80.0) | 53 (98.1) |
| Missing | 0 (0.0) | 0 (0.0) | 0 (0.0) | 0 (0.0) | 0 (0.0) |
| AA = African American  NHB = non-Hispanic Black  NHW = non-Hispanic white  OPI = Other Pacific Islander  *Includes Asian/Native Hawaiian or Other Pacific Islander, unknown, and multi-racial groups.  Bold font indicates significant group differences by race/ethnicity via chi-square test of independence (*p*<.05). | | | | | |
