# Supplementary material for: Predicting salivary cortisol and sexual behavior stigma among MSM in the American Men’s Internet Survey 2019
Source: Sci Rep. 2023 Oct 23;13:18082. doi: 10.1038/s41598-023-44876-z (PMC10593931; doi:10.1038/s41598-023-44876-z)
Supplement: Supplementary file 2 — Supplementary Table S2. [file 41598_2023_44876_MOESM2_ESM.docx]

| **Supplementary Table S2.** Predictive value of sexual behavior stigma (recent) items on various cortisol measures using linear regression among 667 US men who have sex with men, American Mens’ Internet Survey, 2019 | | | | | | | | | | | | | |
| --- | --- | --- | --- | --- | --- | --- | --- | --- | --- | --- | --- | --- | --- |
|  | ***AM Cortisol*** | | | ***PM Cortisol*** | | | ***Average Cortisol*^∞^** | | | ***Daily cortisol change***⸋ | | | |
| **Item** | **Standardized**  ***β***  **(SE)** | **95% CI** | ***p*** | **Standardized**  ***β***  **(SE)** | **95% CI** | ***p*** | **Standardized *β***  **(SE)** | **95% CI** | ***p*** | **Standardized**  ***β***  **(SE)** | **95% CI** | ***p*** |  |
| Excluded from family activities | -.040 (.316) | -.936-.305 | .319 | .039 (.331) | -.328-.971 | .331 | .001 (.229) | -.442-.457 | .975 | -.004 (.335) | -.695-.623 | .914 |  |
| Family made discriminatory remarks | -.043 (.277) | -.835-.253 | .295 | -.025 (.296) | -.765-.398 | .746 | .015 (.169) | -.270-.392 | .718 | -.001 (.245) | -.487-.477 | .983 |  |
| Rejected by friends | -.045 (.429) | -1.33-.353 | .254 | -.007 (.444) | -.952-.792 | .857 | -.036 (.308) | -.886-.324 | .362 | -.026 (.453) | -1.18-.594 | .513 |  |
| Afraid to go to healthcare services | -.040 (.388) | -1.16-.358 | .299 | -.045 (.399) | -1.24-.320 | .246 | -.060 (.277) | -.972-.115 | .122 | -.069 (.408) | -1.53-.071 | .074 |  |
| Avoided going to healthcare services | -.023 (.481) | -1.22-.665 | .562 | -.033 (.494) | -1.39-.550 | .395 | -.039 (.343) | -1.02-.327 | .312 | -.035 (.507) | -1.44-.544 | .373 |  |
| Not treated well in healthcare center | .029 (.912) | -1.12-2.45 | .466 | .004 (.939) | -1.74-1.94 | .917 | .023 (.651) | -.893-1.66 | .554 | -.028 (.959) | -2.56-1.20 | .479 |  |
| Healthcare providers gossiping about you | .003 (.514) | -1.28-1.38 | .942 | -.041 (.704) | -2.11-.648 | .297 | -.027 (.488) | -1.29-.618 | .487 | -.042 (.718) | -2.16-.651 | .291 |  |
| Police refused to protect you | .000 (1.43) | -2.81-2.81 | .999 | -.022 (1.48) | -3.73-2.10 | .584 | -.016 (1.03) | -2.43-1.62 | .697 | -.013 (1.51) | -3.45-2.49 | .751 |  |
| Scared to be in public places | -.024 (.273) | -.701-.371 | .547 | .031 (.280) | -.332-.768 | .436 | .005 (.194) | -.355-.407 | .894 | .027 (.288) | -.363-.768 | .482 |  |
| Verbally harassed | -.047 (.343) | -1.08-.259 | .227 | .003 (.352) | -.661-.720 | .932 | -.030 (.244) | -.668-.291 | .441 | -.028 (.362) | -.974-.449 | .469 |  |
| Blackmailed | .014 (.906) | -1.44-2.11 | .714 | -.021 (.931) | -2.32-1.33 | .597 | -.005 (.647) | -1.34-1.19 | .906 | -.021 (.956) | -2.38-1.36 | .596 |  |
| Ever physically hurt you because MSM | .021 (.616) | -.876-1.54 | .588 | .011 (.634) | -1.06-1.43 | .771 | .023 (.441) | -.603-1.12 | .551 | .027 (.651) | -.823-1.73 | .485 |  |
| Forced to have sex because MSM | .015 (.608) | -.968 - 1.41 | .711 | .017 (.638) | -.970 - 1.53 | .658 | .023 (.440) | -.607 - 1.12 | .559 | .009 (.644) | -1.11 - 1.41 | .810 |  |
| MSM = men who have sex with men  NHW = non-Hispanic white  *β* = coefficient from linear regression for difference in cortisol for those who experienced stigma compared to those who did not; calculated automatically via Stata, version 16.  SE=standard error  ^∞^Cortisol average = AM cortisol + PM cortisol / 2  ⸋Daily cortisol change = \|PM cortisol – AM cortisol\|  “Recent” indicates recently (within the past 6 months) experiencing the sexual behavior stigma item.  Bold font indicates statistical significance (*p*<.05)  Full item descriptions can be found in Table 1. | | | | | | | | | | | | | |
